# Immunotherapy for Diffuse Midline Glioma: From Preclinical Modeling to Clinical Translation

**DOI:** 10.3390/cancers18152411

**Published:** 2026-07-27

**Authors:** Anja Kordowski, Monica Pomaville, Jessica B. Foster, Nicholas A. Vitanza

**Affiliations:** 1 Ben Towne Center for Childhood Cancer and Blood Disorders Research, Seattle Children’s Research Institute, Seattle, WA 98101, USA; 2 Division of Oncology, Children’s Hospital of Philadelphia, Philadelphia, PA 19104, USAfosterjb@chop.edu (J.B.F.); 3 Department of Pediatrics, University of Pennsylvania Perelman School of Medicine, Philadelphia, PA 19104, USA; 4 Department of Pediatrics, Seattle Children’s Hospital, University of Washington, Seattle, WA 98105, USA

**Keywords:** diffuse midline glioma, H3K27-altered (DMG), preclinical models, immunotherapy, clinical trials, pediatric CNS tumors

## Abstract

Diffuse midline glioma, H3K27-altered (DMG) is a group of central nervous system (CNS) tumors with no curative treatment options. First described in the early 20th century, prognosis and clinical outcome for patients have changed very little, and the only standard treatment modality remains radiation therapy. The development of targeted therapies against DMG has long been hindered due to the paucity of tumor tissue and disease models for laboratory research. However, access to patient-derived tumor tissue and the subsequent establishment of DMG preclinical models in recent years has led to a deeper understanding of underlying disease mechanisms and aided the development of novel therapeutic agents. Here, we provide an overview of newly established DMG modeling systems in the preclinical setting leading toward immunotherapy clinical trials for patients with DMG.

## Simple Summary

Diffuse midline glioma, H3K27-altered (DMG) is an aggressive central nervous system (CNS) cancer with a terrible prognosis and no curative treatment options. While radiotherapy remains the standard treatment, an increase in both available patient tissue and dedicated laboratory programs has driven the generation of preclinical models capable of fueling translational research and illuminating tumor biology. In this review, we summarize how preclinical models can be leveraged to assess clinically relevant outcomes for immunotherapies and guide rational clinical trial development.

## 1. Introduction

Diffuse midline glioma, H3K27-altered (DMG) is a highly malignant pediatric diffuse high-grade glioma (HGG) that arises in the midline structures, most commonly the pons, thalamus, and spinal cord [1]. DMG, which, if located in the pons, is often colloquially referred to as diffuse intrinsic pontine glioma (DIPG), equally affect males and females between 4 and 15 years of age. The median overall survival (OS) is less than 1 year and more than 90% of the children die within 2 years [2,3]. In the US, >300 children are diagnosed with DMG annually, which correlates to about 50 children in the UK or 20 patients in Australia.

Dismal prognosis and short life expectancy can partially be attributed to the tumor’s location. Due to critical biological functions of the midline structures in the brain, substantial surgical resection is often not an option, and biopsy procedures were historically rare. As a consequence, access to tumor tissue was sparse and biologic understanding of the disease was limited. Given the lack of information on molecular and biological features, the World Health Organization (WHO) previously grouped these tumors with their adult counterparts and the choice of drugs for clinical trials was often based on experience in adult HGG [4,5]. However, in the early 2000s, data began to emerge that stereotactic biopsies were feasible and often tolerable for children with DIPG/DMG [6]. Around the same time, the first tumor cell cultures and xenograft models were generated from patient autopsy tissues [7]. This success was followed by global increase in patient tumor donations which mobilized wider access to tumor autopsy and treatment-naive patient samples for laboratory research.

The subsequent onset of preclinical model development, together with concomitant advancements in sequencing technologies, has revolutionized our understanding of DMG biology and targeted therapeutics. Seminal publications in 2012 described the global loss of histone 3 (H3) trimethylation, a hallmark of DMG [8,9,10]. H3 hypomethylation is primarily caused by a lysine27 (K27) mutation in the histone H3 genes *H3F3A* or *HIST1H3B*/*C* affecting the histones H3.3 or H3.1, respectively. To a lesser extent, EZH inhibitory protein (EZHIP) overexpression is found in H3-wildtype tumors, likewise leading to polycomb repressive complex 2 (PRC2) inhibition and, ultimately, global loss of H3K27 trimethylation [11]. As a result of this rapid information gain, the 2016 edition of the WHO Classification of Central Nervous System Tumors first separated diffuse glioma in children from adult gliomas. The 2021 edition then refined this group to DMG, H3K27M-mutant, which was further changed to DMG, H3K27-altered [5,12]. The advancing field of single-cell and spatially resolved sequencing as well as immunohistochemical (IHC) staining of patient tissues and tumor models was further critical to examining the tumor immune microenvironment (TIME). Elucidating the immune biology of these tumors specifically sparked wide interest in the field. A better understanding of the TIME and utilizing DMG tumor models allows for trialing different immunotherapy modalities in the preclinical setting and for assessing the application of such approaches. For instance, studies from adrenocortical carcinoma and bladder cancer showed that integrating the role of the TIME may identify patients that respond to immunotherapies against these malignancies [13,14].

While the disease has remained without a cure, and the standard-of-care treatment is still limited to radiation therapy, the first therapeutic for progressive thalamic DMG was approved by the FDA in 2025, stemming from years of preclinical research with dordaviprone [15]. DMG models are now indispensable tools, not only to study biology but for testing novel, disease-specific therapeutics that can be brought to patients and then back to the lab for iterative enhancements. This review summarizes how models of DMG advance the scientific translation of more efficacious treatment strategies for patients, with a particular focus on current immunotherapy approaches.

## 2. DMG Modeling Systems

Models of DMG are generated from patient-derived tissue or are genetically engineered to create cell cultures, organoids, and animal model systems [16]. Patient-derived tissue has been instrumental in exposing inherent biology but faces limitations due to the standard requirement for an immunocompromised host and cross-species immune restrictions. However, the DMG tumor microenvironment and host immune system is a critical component of tumor activity and therapeutic response. Therefore, model selection is central to explaining the abilities and shortfalls of one’s study, and a wide array of model systems allow a multifaceted approach to test the efficacy of immunotherapies. A brief overview of model systems and their application to test immunotherapies is described here.

### 2.1. Patient-Derived Tissue Models

Collaborative efforts to systematically collect patient-derived tumors for research from organizations such as Gift From A Child and the Children’s Brain Tumor Network (CBTN) has enabled the establishment of models for the study of new therapies [17]. Investigators have relied heavily on such patient-derived tissue to examine the antitumor effects of immunotherapies [18,19,20,21,22,23,24].

#### 2.1.1. Cell Culture Models

Cells have historically been collected from post-mortem tumor resection, and—upon the discovery that biopsy at the time of tumor diagnosis is both safe and feasible for patients with DMG—primary human DMG cells from treatment-naïve tumors have been used to test immunotherapies. Patient-derived orthotopic xenograft models have been frequently used to model DMG, both direct from patient tumor tissue or from patient-derived cell models implanted into anatomically relevant brain structures. Cells derived directly from patient tumor tissue have variable growth and engraftment patterns in mice, and if taken from patient to mouse directly have limited ability to insert reporter genes. To overcome this challenge, multiple human DMG models have been found to grow in culture with reliable growth kinetics [21,25]. SU-DIPG-IV (H3.1 subtype), SU-DIPG-VI (H3.3 subtype), and SU-DIPG-XIII (H3.3 subtype) were some of the earliest autopsy-derived models and were transformative for laboratory DIPG research [26]. More recently, biopsy-derived models, such as PBT-22FH, PBT-24FH, PBT-27FH, PBT-29FH, 7316-6349, 7316-3058, and SF8628 cells have been generated at multiple institutions [19,27,28,29]. Although this does not represent a complete list of all available models, the independent establishment at multiple locations highlights the feasibility and utility of direct-from-patient research modeling. Some of these models can be stereotactically injected into the mouse pons and result in rapid tumor formation with subsequent tumor-related death, providing a critical system for rapid therapeutic testing. Patient-derived tumors are most often implanted into the CNS of immunocompromised mice (e.g., NOD/SCID or NSG), which does limit comprehensive examination of tumor:host interactions due to the scant immune cells present [30,31]. Additional model systems include zebrafish, which share many conserved organ systems with humans [32] and patient-derived U-87 glioblastoma cells engineered to express H3.3K27M [33].

#### 2.1.2. Organoid Models

Organoids derived from DMG tumors at tumor biopsy provide a more complex (e.g., containing supportive structures and microglia), if not complete, ex vivo system to evaluate therapeutic efficacy and may often be derived from patients prior to treatment [19]. However, there are inherent limitations as there are frequently missing components, such as astrocytes and myeloid cells—whose differentiation is challenging to control in vitro—so their utility may be in answering particular biologic questions rather than bridging in vitro and in vivo efficacy studies. Placing organoids into immunocompromised mice may carry potential to more closely replicate the environment of DMG cells; however, the reproducibility of patient-derived organoids in culture over time has not been fully validated [34]. To emulate a more complex cooperation of tumor cells with their environment, co-culturing methods involving DMG cells together with immune cells such as macrophages have revealed interaction patterns that reflect immunoregulatory phenotypes such as cytokine production [30]. Other co-culture systems that have been used previously to study DMG infiltrative characteristics include DIPG:neural assembloids. Human-induced pluripotent stem cells (hiPSCs) were utilized to establish neural organoids, which were further co-cultured with DIPG spheroids [35]. Other groups established fluorescently labeled single-cell model systems, comprising DMG tumor cells, human brain microglia, and endothelial cells. When co-cultured, the cell types were shown to self-assemble and to generate 3D multicellular tumor tissue-like aggregates, termed tumor tissue analogs (TTAs) [36].

### 2.2. Engineered Model Systems

To overcome inherent limitations present in patient-derived models, engineered systems have helped to generate models that allow for therapeutic testing in a more immunocompetent setting, or for an evaluation of therapies in reproducible genetic settings [16]. Cell culture and animal model systems have each been employed to generate such models for the evaluation of immunotherapies.

#### 2.2.1. Stem Cell Models

Engineered stem cell models have been generated to recapitulate the primary molecular features present in DMG tumors [37]. Early neural progenitor cells (NPCs) derived from human embryonic stem (hES) cells form cells reminiscent of DMG following the co-expression of H3.3K27M and mutant PDGFRA, and suppression of p53. These cells grow in the brains of immunocompromised mice that recapitulate growth patterns of human DMG. De novo methods of DMG formation have also been applied to organoid systems, where lineage-specific cell types were developed by a morphogen system that cooperated with multiple cell types in the DMG microenvironment [38]. Similarly, methods to induce differentiation of stem cells into immune cells could be used to generate replicable components of immune–tumor interactions.

#### 2.2.2. Genetically Engineered Animal Models

Genetic engineering approaches use viral (e.g., lentiviral, retroviral, or adeno-associated virus vectors) and non-viral (e.g., sleeping beauty transposons, Cre/lox, PiggyBac transposons, or electroporation) methods to produce genetically engineered mouse models (GEMMs) [16]. In utero electroporation (IUE), Cre/lox, and the RCAS-t/va system are example systems used to engineer syngeneic mouse models to recapitulate DMG [39]. Despite the inherent limitations in reflecting the complete genetic profile of human DMG, they are often designed to possess an intact immune system. GEMMs have been used to replicate *H3.1K27M* and *H3.3K27M* mutant DMG tumors with various concomitant genetic aberrations that spontaneously develop tumors with different latency periods and locations to resemble human DMG [40,41,42].

IUE was used to introduce histone mutations together with molecular aberrations typical to DMG tumors (e.g., *ACVR1*, *FGFR1*, *NF1*, *PDGFRA*, *PIK3CA*, *PPM1D*, *TP53*) [40,42]. Although K27M was unable to drive tumor development alone, one additional mutation, such as *TP53* loss, *NF1* loss, or *FGFR1* mutations, allowed for tumor development. Cells taken from these models were transplantable into immunocompetent mice and could then be used for therapeutic testing and to identify biological features of DMG tumors and their microenvironment. Multiple models of both immunocompetent and immunocompromised mice were used to dissect the activity of individual microenvironment components and their correlation to human DMG profiles [40]. The tumor microenvironment was evaluable, to some degree, in many models, showing myeloid infiltration (diverse cell types, including microglia) that helped to sustain tumor formation and maintenance of H3.3K27M syngeneic mouse models. The 4-hit de novo tumor model, which leverages multiple concomitant genomic aberrations to form tumors in C57BL/6J mice, showed intratumoral and microenvironment features that reflect characteristics of human tumors, providing a model that may be useful in the evaluation of response to immunotherapies. The RCAS/tv-a avian retroviral system has been used to develop brainstem glioma models [43,44,45]. Similar to models generated by IUE, multiple genomic events, such as overexpression of *PDGFB* and *TP53* abrogation, were required to most closely replicate sustainable growth patterns. An RCAS/tv-a avian retrovirus system has also been combined with CRISPR/Cas9 genetic engineering to induce DMG formation in mice [45].

Syngeneic zebrafish models may also supplement murine models and carry the advantages of efficient tumor modeling, therapeutic response assessment, and variable levels of immune response evaluation [46]. These also can be used in the interpretation of microglia:DMG interactions.

#### 2.2.3. Naturally Occurring Gliomas

Although large animals may better recapitulate human anatomical and immunological features, they are not routinely used for the evaluation of immunotherapy efficacy due to ethical considerations regarding inducing tumors. However, naturally occurring gliomas form in certain species, such as canines, which can be used to examine molecular glioma properties, assess interactions with host environment, and evaluate novel therapeutics [47]. For example, tumor-encoding mRNA antigens were formulated in multi-lamellar lipid particle aggregates (LPA) that functioned as vaccines and as immunomodulating agents [48,49]. This therapy was evaluated in canines with end-stage glioma and showed remodeling of the TIME (i.e., increased gene signatures for antigen presentation, IFN signaling, and cytotoxicity) and signals of prolonged survival in comparison to historical survival trends. Spontaneous gliomas that form in canines do not contain H3K27M mutations and may more closely resemble histone wild-type HGG; however, there may be other naturally occurring gliomas that could be used to study DMG. Gene transfer has been successfully completed in canines and could represent an additional method of in vivo genetic manipulation [50].

### 2.3. Preclinical Evaluation of Immunotherapies

A culmination of cell culture, organoid, immunocompromised, and immunocompetent animal models are used to evaluate the preclinical efficacy and potential toxicity of immunotherapies [51]. Selection of the appropriate model is dependent on the underlying question.

#### 2.3.1. Ex Vivo Approaches

Patient-derived cell and organoid models are used for multiple functional assays. Standard assays such as cell viability, flow cytometry for cell surface protein expression, markers of apoptosis, and cytokine assays are carried out in these cells for the efficient early evaluation of immunotherapies. Both can be sorted into single-cell suspension to analyze cell surface expression patterns. Interestingly, cells grown in a monolayer versus cells grown as neurospheres exhibit distinct genomic and physical characteristics, such as an altered extracellular matrix, which may affect the study of immunotherapies and selection of assays [16,52]. Like tissue, organoids may be sliced into sections for immunofluorescence analysis and stained with markers to characterize therapy response, such as T cell infiltration and apoptosis [19]. DMG models have also been exposed to natural killer (NK) cells, T cells, and healthy donor macrophages [30]. In this system, in isolation, only NK cells were able to induce tumor cell lysis.

#### 2.3.2. In Vivo Approaches

Animal models allow for the testing of immunotherapies in a more complex cellular and structural environment. They have been used to evaluate the anti-tumor activity, tumor:host biological effects, and kinetic patterns of therapies. The establishment of in vivo approaches to study immunotherapies has allowed for the direct clinical translation of novel immunotherapies, as in the case of introduction of B7-H3 chimeric antigen receptor (CAR) T cells as repetitive intracranial injections to children following establishment of safety in mice [53]. Oncolytic viruses, such as oncolytic herpes simplex virus and mesenchymal stem cell-carrying oncolytic viruses have been tested in multiple types of animal models to evaluate anti-tumor efficacy [51]. Kinetic evaluations of immunotherapies, such as CAR T therapies, have shown diffuse penetration into CNS structures following injection into mice. Labeled disialoganglioside (GD2) CAR T cells showed diffuse spread across the leptomeninges of treated mice by day 7 post-injection, and, by day 14, post-injection cells infiltrated brain parenchyma with many foci of microglia and markers of apoptosis [22]. Conversely, CD19 CAR T cells, frequently used as a negative control comparison, did not show similar levels of infiltration. The transient properties of mRNA CAR T cells were also demonstrated, showing a presence lasting approximately 9 days [19] and efficacy with repeated dosing in murine xenograft models. Although the use of immunocompetent animal models is useful to examine biological questions such as tumor–host interactions, the divergent sequence homology between mouse and human T cells that necessitates the generation of murine CAR T cells adds a layer of complexity to analysis and may not fully recapitulate the corresponding human construct [54]. Mechanical differences in therapy delivery between humans and mice may further complicate the interpretation of preclinical experiments [55].

#### 2.3.3. Toward Combination Therapies

Looking ahead, patients with DMG may inevitably require the addition of therapies that can synergize with immunotherapies to achieve a durable response to therapy. Evaluation of immunotherapies using multiple methods allows for thoughtful combination development upfront. For example, an oncolytic virus that was encapsulated in mesenchymal stem cells (CRAd.S.pK7) reduced cell viability in preclinical models [56]. In the in vivo setting, only combination with radiation therapy led to a meaningful prolongation of survival compared to oncolytic virus alone. In this system, intranasal administration was also compared to local administration to the CNS and showed that the delivery of CRAd.S.pK7 directly to local tumor site versus intranasal administration led to anti-tumor effects in mice. Epigenetically targeted therapies such as histone deacetylase (HDAC) inhibition have been used to show antitumor activity with NK cell therapy in orthotopic mouse models [57]. Immune checkpoint blockade using an aptamer was combined with radiation therapy in preclinical models [58]. CAR T cell therapies also have been combined with multimodal types of therapy in preclinical models. For example, the small molecule imipridone ONC206 was dosed together with B7-H3 CAR T cells, enabling lower E: T ratio of CAR T cells to achieve antitumor effect [24]. Oncolytic viruses have been used to boost both B7-H3 and GD2 CAR T cell anti-tumor response without affecting CAR T cell viability and while inducing enhanced cytokine release [59]. Identification of synergistic therapy combinations in preclinical models like this may provide patients with more durable antitumor effects from immunotherapy.

## 3. Clinical Immunotherapy Trials

As described above, preclinical modeling of the DMG TIME is key to predict immunotherapy response and pave the way for clinical translation. From an immunological perspective, DMG is often considered “cold” or “inert”, characterized by low mutational burden and a paucity of infiltrating T lymphocytes but elevated immunosuppressive myeloid populations. An early radiogenomic study combining imaging characteristics from 28 DMG patients and correlated RNA sequencing results from matching tissue specimens at autopsy classified these tumors into three subgroups: lymphocyte-depleted, immunologically quiet, and inflammatory [60]. While there is no standard for how to immunologically stratify patients for clinical trials, DMG likely exhibits an immunologically complex environment that, while marked by a low inflammatory signature, may be predicted to be responsive to immunotherapies, though barriers such as the myeloid compartment exist [30,31,61]. The recent explosion of clinical immunotherapy approaches against these tumors has dovetailed with a growing understanding of the DMG TIME [62]. Iterative studies can now aim to improve our preclinical testing and, in concert, enhance the clinical efficacy of future clinical trials. Here, we outline the spectrum of immunotherapy strategies that are shaping the clinical trial landscape for children and young adults with DMG.

### 3.1. Immune Checkpoint Inhibitors

Immune checkpoint inhibitors (ICIs), including cytotoxic T-lymphocyte-associated protein-4 (CTLA-4), programmed cell death protein-1 (PD-1) and PD-1 ligand (PD-L1) inhibitors, have emerged as a promising immunotherapy against a range of cancers [63]. This class of inhibitors largely functions via lymphocyte regulation and reactivating anti-tumor response by blocking inhibitory T cell signaling [64,65]. ICIs demonstrated efficacy against adult cancers and some pediatric cancers, including pediatric hepatocellular carcinoma and Hodgkin lymphoma [66,67,68]. A study evaluating response to PD-1 blockade in children with progressive or relapsed hypermutant tumors associated with germline DNA mismatch repair deficiency (MMRD) showed durable responses and improved survival [69]. The corresponding study (NCT02992964) assessed the PD-L1-targeting monoclonal antibody (mAb) nivolumab, showing benefits in pediatric patients with high tumor mutation burden [70]. However, checkpoint blockade shows only limited efficacy in pediatric brain tumors, which is largely attributed to the low neoantigen burden, low checkpoint marker expression, and limited T cell presence [30,31]. In a phase 1 clinical trial, nivolumab was tested alone and in combination with the CTLA-4-targeting mAb ipilimumab in pediatric patients with CNS tumors, including DMG (NCT03130959). Whilst treatment was considered safe, neither study showed improved survival over radiotherapy alone in DMG patients [71]. A small subset of pontine HGG may be classified as hypermutant and can occur in the setting of mismatch repair syndromes, in which case they could be predicted to have superior responses to ICI.

### 3.2. Neoantigen Vaccines

Vaccine-based strategies against DMG have shown early promise but challenges remain. Cancer vaccines, divided into peptide vaccines and dendritic cell vaccinations, are designed to induce a durable anti-tumor immune response. Tumor-associated/-specific antigens (TAAs/TSAs) are administered and processed by antigen-presenting cells, which are then presented to immune cells, specifically T cells [72]. A phase 1 clinical trial evaluated the safety of a short 10-mer peptide vaccine directed against the H3.3K27M protein in patients with HLA-A*02:01 and H3.3K27M-positive DMG tumors (NCT02960230). Although there are differing conclusions on H3.3K27M as an HLA-A*02:01-binding epitope, patients with a disease response had H3.3K27M-reactive CD8+ T cells [73,74]. The median OS for patients with immunological response was 16.3 months compared to 9.9 months for non-responders [75]. Similarly, a first-in-human phase 1 trial evaluating a longer 27-mer vaccine, encompassing the H3K27M_p14-40_ epitope, alone or in combination with anti-PD-1 therapy in adults with DMG, was shown to be safe and induced CD4+ T cell immune responses, and one patient showed complete remission over 31 months of follow-up [76]. These early promising results warrant further investigation of developing vaccine-based immunotherapies against DMG. However, due to a largely immunosuppressive TIME, cancer vaccines as a monotherapy against these tumors may be ineffective, and current ongoing trials explore combination therapies. Two current trials include a dendritic cell vaccination tested together with temozolomide-based chemoradiation, as well as rHSC-DIPGVax, an off-the-shelf peptide-based vaccine directed against recombinant human heat-shock proteins tested in combination with checkpoint blockade.

### 3.3. Oncolytic Virus Therapy

Oncolytic immunovirotherapy is another developing field with promising therapeutic potential against DMG. This form of anti-cancer therapy utilizes a virus’ inherent ability to selectively infect and lyse a cell. A further advantage of this mechanism is that the virus may stimulate pro-inflammatory signals, thereby provoking responses from the adaptive immune system. The oncolytic adenovirus DNX-2401 was effective against pediatric glioma and DIPG in preclinical mouse models and led to a clinical trial [77]. The following phase 1 trial tested the intratumoral infusion of DNX-2401 for newly diagnosed patients with DIPG (NCT03178032). Results demonstrated reduction or stabilization of disease for some patients, and an examination of patient autopsy tissue confirmed alteration in the TIME, such as T cell activation status. However, treatment was also associated with adverse side effects, including hemi- and tetraparesis in two out of 12 cases [78]. Other studies investigate the efficacy of additional interleukin (IL)-expressing adeno vectors to specifically modify the TIME and to generate a pro-inflammatory environment. Current trials testing this potential are the adenoviral vector-based Ad-TD-nsIL12 study for primary or progressive DMG [79], as well as AloCELYVIR, a therapy that utilizes bone marrow-derived mesenchymal stem cells for delivery. Opportunities to use an IL-12-expressing herpes simplex virus-1 (HSV-1), M032, against newly diagnosed patients with DMG, are also currently being explored (NCT07076498).

### 3.4. CAR T Cells and Other Adoptive Therapies

CAR T cell therapies have revolutionized the treatment of hematological malignancies and currently represent the most encouraging form of targeted cellular therapy against DMG following landmark phase 1 clinical studies of B7-H3 and GD2 CAR T cells [53,80,81]. BrainChild-03, a phase 1 clinical study of intracerebroventricular (ICV) B7-H3 CAR T cells for patients with recurrent/refractory CNS tumors and DMG was the first CAR T cell trial to inject CAR T cells intracranially to a patient with DIPG via ICV administration (NCT04185038) [81]. Overall survival (OS) from diagnosis for all treated patients with DIPG was 19.8 months, with three patients still alive more than four years from diagnosis. ICV B7-H3 CAR T cells resulted in one dose-limiting toxicity of intratumoral hemorrhage, though common toxicities were clinically manageable fever, headaches, and nausea [81]. While encouraging, an upcoming multi-site phase 2 pivotal registration trial is necessary to define the clinical efficacy of these CAR T cells. Similarly, promising results were observed with GD2 CAR T cells for pediatric patients with DMG (NCT04196413) [80]. Four of 11 patients that received ICV-injected GD2 CAR T cells showed substantial reduction in tumor volume, with one patient demonstrating sustained complete response. Treatment-related adverse events and toxicities were observed but generally manageable with specialized care. Other current CAR design strategies include a range of enhancements, including the co-targeting of multiple antigens to overcome treatment barriers such as antigen escape and tumor heterogeneity. For example, there is an ongoing first-in-human phase 1 trial delivering CAR T cells co-targeting B7-H3, EGFR806, HER2 and IL13-zetakine (NCT05768880) for children with DIPG/DMG, as well as refractory CNS tumors. Importantly, while there have been clinical successes in CAR T cell clinical trials to-date, responses are not universal and improvements in potency will require better immunocompetent preclinical modeling to understand potential corresponding increases in toxicity. Further adoptive cellular therapies that are currently being explored for the treatment against DMG include anti-H3.3K27M T cell receptor-expressing T cells and utilizing natural killer (NK) cells. The former will determine the safety and tolerability of intravenous-injected T cells in HLA-A*0201-positive DMG patients, whereas two early-phase clinical trials are investigating the safety and efficacy of NK cell transplants.

Microglia replacement and engineering also offer novel strategies with potentially therapeutic benefits against DMG. Groups previously demonstrated successful depletion of brain-resident microglia via colony stimulating factor 1 receptor (CSF1R) inhibition [82,83,84]. Subsequent bone marrow transplantation and microglia replacement showed therapeutic efficacy against various neurologic diseases [85,86,87,88].

Lastly, improving delivery routes for immunotherapies to bypass the blood–brain (BBB) and blood–brain tumor barrier (BBTB) could be a way to achieve better anti-tumor responses. New technologies have mainly focused on convection enhanced delivery (CED), focused ultrasound (FUS), and intrathecal administration via Ommaya reservoirs [89,90]. Different immunotherapy avenues show early promising clinical benefits. Achieving universally durable responses remains challenging due to the unique features of the disease and notably, the pediatric TIME. A multimodal treatment approach that combines different treatment strategies will therefore likely be required.

## 4. Clinical Correlates

To establish the fidelity of preclinical models to human counterparts in terms of efficacy and toxicity, a thorough analysis of preclinical model outcomes following immunotherapy administration should be compared with correlative studies that emerge from clinical trials. Correlative analysis has included examination of clinical signs and profiling of serum, cerebrospinal fluid (CSF), and tissue [53,80,81].

### 4.1. Clinical Correlates to Preclinical Models

BrainChild-01, a phase 1 clinical trial evaluating the repetitive dosing of HER2 CAR T cells to children and young adults with recurrent or refractory CNS tumors (NCT03500991), described local CNS immune activation following intracranial CAR T cells for children, evidenced by high concentrations of chemokines and cytokines (e.g., CXCL10 and IFNγ) in the CSF [91]. This same pattern was observed following B7-H3 CAR T cells, in which a substantial elevation in T cell pathway cytokines representative of CAR T activation (e.g., IFNγ) and T cell trafficking (e.g., CXCL10) was observed, suggesting cytotoxic anti-tumor effect signals [53,81]. Based on these findings, a preclinical study of B7-H3 CAR T cells genetically modified to overexpress chemokine receptor CXCR3-A demonstrated elevated CAR T cell trafficking and efficacy against DMG compared to standard B7-H3 CAR T cells [23]. Adjustments in cytokine profiles seen following CAR T treatment are often concomitant with periods of neuroinflammation and may be a general marker of immune reaction to therapy. The purpose of many of these cytokines in the context of immunotherapy remains to be fully elucidated, and models that more closely replicate host–tumor interactions may help to dissect these roles.

In patients treated with lentiviral GD2 CAR T therapy, cytokine elevation patterns corresponded with site of administration; cytokine elevations in CSF were seen with ICV injection, and cytokine elevations in plasma were seen with IV administration [80]. CSF levels of immunosuppressive TGFβ factors increased following GD2 CAR T administration in some patients, and higher TGFβ1 levels early after GD2 CAR T infusion trended with disease progression. In contrast, TGFβ levels were low in the CSF of one patient who had a complete response to therapy. Certain cytokines (e.g., CCL2) correlated with more severe presentations of tumor inflammation-associated neurotoxicity (TIAN) [92]. Digital droplet PCR was also used to detect H3K27M mutations from CSF, and, in some patients, a decrease was associated with tumor regression, while there was an increase at times of peak inflammation or disease progression. The interaction between DMG organoids and microglia has also been evaluated, including cytokine expression (e.g., IFNγ) [38]. Table 1 shows examples of how preclinical immunotherapy studies informed subsequent clinical trials.

### 4.2. Modeling Neurotoxicity

Murine models have largely accounted for preclinical studies evaluating the toxicity of immunotherapies. The orthotopic stereotactic-guided injection of DMG cells into anatomically relevant regions of the mouse brain, revealing location-specific toxicity patterns. GD2 CAR T cells injected into mice with thalamic DMG tumors caused neurotoxicity [19,22]. In immunocompromised mouse models, neuropathological evaluation of resected mouse brains showed ventricular enlargement that represented hydrocephalus, and surrounding inflammation of brainstem parenchyma that were both proposed to represent toxicity profiles consistent with antigen-specific antitumor activity [22]. This can potentially be mitigated through the use of transient mRNA CAR T cells targeting GD2, where dose-adjusted treatment against diffuse midline glioma murine models maintained efficacy while sparing neurotoxicity. Notably, some immunotherapies tested in model systems (i.e., B7-H3 CAR T cells in immunocompromised mouse models) for IND-enabling studies have not shown neurotoxicity, which has been encouraging [53]; however, toxicity can vary based on the therapy and model, so this data should not be interpreted as the complete lack of toxicity. Rather, the rich spectrum of models now available allows for specific biological investigations using the best available model to interrogate specific questions.

Early findings suggest that there may be long-term neurocognitive difficulties with attention and memory following CAR T therapy, and cognitive impairment is a known long-term effect of anti-cancer therapy. Following GD2 CAR T cell-mediated tumor clearance in NSG mice with implanted SU-DIPG-VI DMG cells to the pons, there was evidence of impaired cognition, microglial reactivity, and persistent cytokine elevation (e.g., CXCL10, CCL2, CCL7, CCL11) [93]. These experiments represent ways in which neurocognitive and correlative data may both be used to holistically evaluate the effects of immunotherapy.

**Figure 1:**
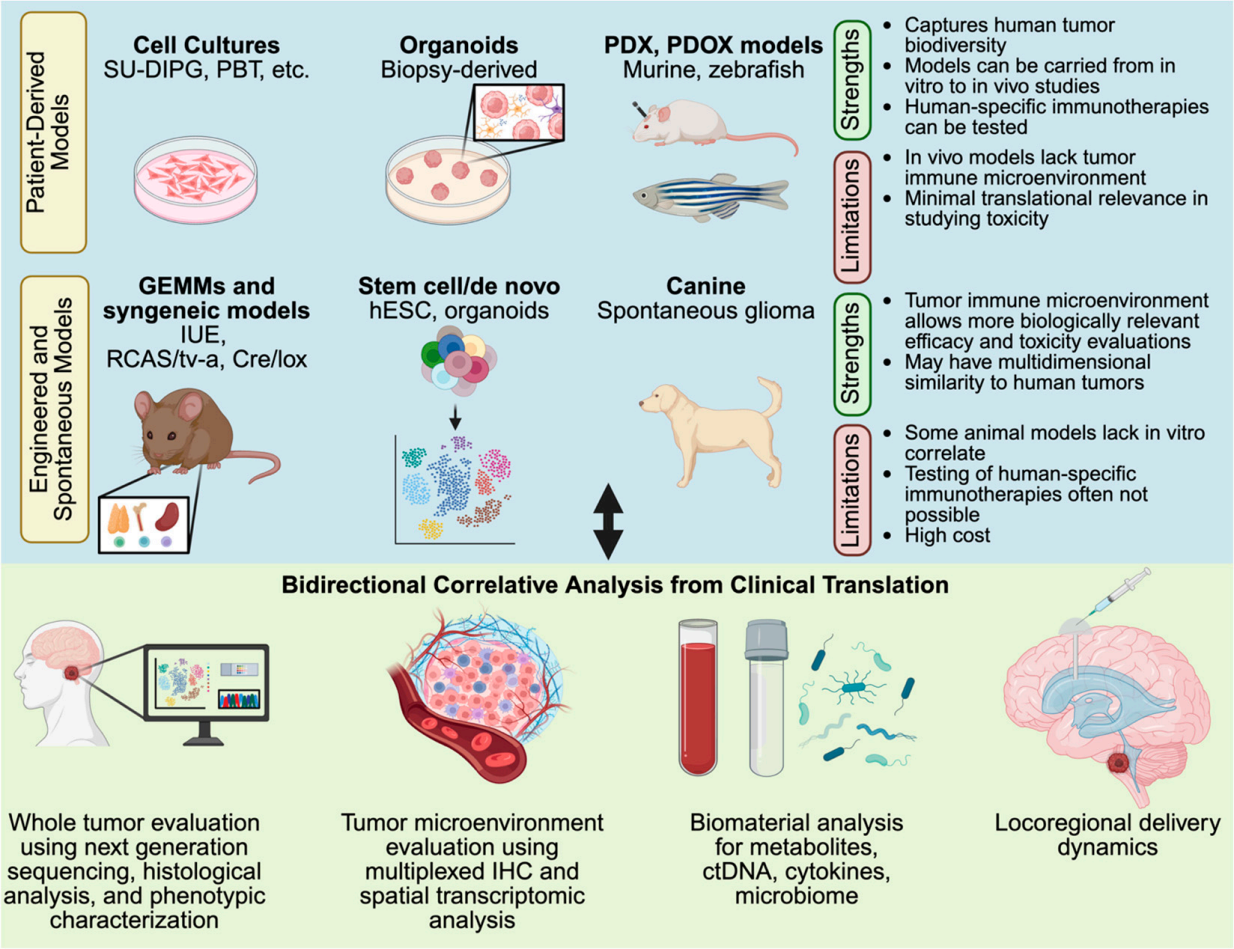
Application of preclinical models for clinical translation of immunotherapies for diffuse midline glioma. Created in BioRender. Pomaville, M. (2026) https://BioRender.com/okvwy1r (accessed on 16 July 2026). Abbreviations: SU-DIPG: Stanford University–diffuse intrinsic pontine glioma, PBT: pediatric brain tumor, PDX: patient-derived xenograft, PDOX: patient-derived orthotopic xenograft, GEMM: genetically engineered mouse model, IUE: in utero electroporation, RCAS/tv-a: Replication-competent avian sarcoma/tumor virus receptor A, Cre/lox: Causes recombination/locus of crossover, hESC: human embryonic stem cell, IHC: immunohistochemistry, ctDNA: circulating tumor DNA.

Figure 1 highlights examples of preclinical models and bidirectional correlative analysis that can drive iterative improvement of immunotherapy trials for children and young adults with DMG.

**Table 1:** Examples of preclinical reports to early clinical findings for some immunotherapy trials.

Immuno- Therapeutic	Preclinical Models	Preclinical Results	Clinical Results
H3K27M-vac (vaccine)	In vitro: patient-derived cell cultures (e.g., HSJD-DIPG-017, U87); In vivo: orthotopic xenografts (NSG, MHC-humanized murine models)	H3K27M-targeted vaccine induced efficient CD8+ T cell-mediated tumor cell killing in vitro and suppressed glioma progression in vivo [33,94].	(A) NCT02960230; (B) No grade-4 treatment-related adverse events. Treatment was well tolerated; (C) median OS was 16.1 months for patients with H3.3K27M-reactive CD8+ T cell responses [75]. (A) NCT04808245; (B) no DLTs; (C) Median OS was 12.8 months following immune response; one patient showed sustained remission for >31 months; (D) treatment-induced CD4+ T cell responses [76].
DNX-2401 (oncolytic virus)	In vitro: patient-derived cell cultures (e.g., TP54, TP80, JHH-DIPG1, SU-DIPG IV); In vivo: orthotopic xenografts (NSG and BALB/c), syngeneic (e.g., NP53, XFM)	Delta-24-RGD efficiently replicates and shows antitumor efficacy. Extended survival in patient-derived orthotopic models of DIPG/pHGG and immunocompetent syngeneic models [77].	(A) NCT03178032; (B) No grade 4 or 5 events; (C) Median OS was 17.8 months. One patient was free of tumor at 38 months of follow-up. Reduction in tumor size was reported in 9 patients, partial response in 3 patients and stable disease in 8/12 patients; (D) Alterations in TIME and T cell repertoire were observed in one autopsy sample [78].
B7-H3 CAR T cells	In vitro: patient-derived cell cultures (e.g., PBT-29, SU-DIPG-6, U87); In vivo: orthotopic xenografts (NSG)	B7-H3 CAR T cells mediate cytokine production and cell lysis of CNS cancer models in vitro. Extension of survival in a panel of orthotopic xenograft models [53,95].	(A) NCT04185038; (B) One grade 4 DLT, most treatment-related adverse events were grade 1 and 2; (C) median OS for patients with DIPG was 19.8 months. Three patients were still alive 44.6, 45.6 and 52.5 months from diagnosis at time of report [81].
GD2 CAR T cells	In vitro: patient-derived cell cultures (e.g., SU-DIPG-6, VUMC-DIPG10, QCTB-R059, SU-pSCG); In vivo: orthotopic xenografts (NSG)	GD2 CAR T cells mediate cytokine production and potent cell lysis in vitro. Showed significant efficacy in patient-derived orthotopic xenograft models of DIPG/DMG. Neurotoxicity seen in thalamic DMG xenograft models [22].	(A) NCT04196413; (B) CRS was observed following IV GD2 CAR T cells. No DLTs were described for ICV administration of GD2 CAR T cells; (C) median OS for DIPG patients was 17.6 months and for spinal DMG 31.9 months. Two patients were still alive at time of data cut-off (33- and 30-months of follow-up) [80].

## 5. Conclusions

DMG remains a therapeutically intractable cancer. Immunotherapies against these malignancies show promise; however, distinct tumor-intrinsic features of DMG and an unfavorable TIME hamper more durable treatment responses. Hence, better characterization of the TIME and identification of biomarkers will be decisive for predicting therapy responses. Conforming to this, a comprehensive study highlighted that the immune landscape of pediatric CNS tumors can be a key prognostic factor and a determinant for personalized treatment strategies [96]. Similarly, post-treatment sampling and representative models provide invaluable insights into potential treatment resistance mechanisms and biological changes [97]. Ongoing studies will contribute to our understanding of the heterogeneity that exists within the TIME to better guide tailored treatment approaches.

To date, the effort of patients, patient advocates, and investigators has driven robust efforts to centrally collect biomaterials and molecular characterize tumors from patients with DMG, enabling open access to these precious resources. These initiatives will support downstream multidimensional analysis of patients treated with immunotherapies, providing a path to improve therapies in the future.

Preclinical modeling is essential to migrate therapies to patients. While no single preclinical model is universally superior, each model’s strengths and limitations should inform what model is chosen for any particular study. Antigen target discovery, tumor genetics and epigenetics, and screening of therapeutics may be rigorously performed using patient-derived in vitro studies. Organoids may answer specific biologic questions as conditions can be controlled, even if they are not entirely biologically faithful. In vivo, emerging immunocompetent models such as C57BL/6 mice that undergo IUE of genomic aberrations that mimic DMG are sparking new understanding of the interface of immunotherapy and the TIME. While clinical trial stratification for patients with DMG based on their TIME is currently not applied, integrating characteristics of this is an important strategy to predict patients’ responses to immunotherapy, ultimately guiding personalized treatment regimes. Clinical trial enrollment for DMG patients is, at present, either very specific or too universal. Accordingly, preclinical modeling of individual patient tumors and testing therapeutic responses may better predict a more tailored clinical trial enrollment or, conversely, help to better subgroup patients for clinical trial design. Hence, orthotopic xenograft models may still provide first-pass assessments and fuel clinical translation. However, it should be considered that any one model is unable to fully predict therapeutic efficacy or toxicity. Ultimately, the utilization of multiple model systems will be necessary to characterize novel immunotherapies. In a regulatory setting with the goal of developing methods of studying both toxicity and tumor response to therapy that do not rely on animal models, novel de novo tumor models need further exploration. Emerging methods of toxicity screening that replicate three-dimensional biophysical properties of tumors with co-cultured immune cells may help fill these gaps and reduce the number of animals needed per study. In addition to studying tumor-intrinsic responses to therapy, a specific examination of cells in the tumor microenvironment is critical to understanding response to immunotherapy.

Finally, correlative analyses of biospecimens derived from patients treated during clinical trials is critical to our understanding of response to immunotherapy. This will not only characterize the efficacy and toxicity of each therapy but will also shed light on the integrity of model systems to reproduce effects seen in humans, providing a path forward as gaps in knowledge become known. A closed-loop approach beginning with efficient testing of efficacy and safety in rationale laboratory models will hasten clinical translation. Then, robust correlative studies can inform our next generation of preclinical experiments to optimize these therapies and, ultimately, pave an iterative path towards cures.

## Data Availability

No new data were created or analyzed in this study. Data sharing is not applicable to this article.
